# Comparison of breast cancer metastasis models reveals a possible mechanism of tumor aggressiveness

**DOI:** 10.1038/s41419-018-1094-8

**Published:** 2018-10-10

**Authors:** Nir Pillar, Avital Luba Polsky, Daphna Weissglas-Volkov, Noam Shomron

**Affiliations:** Sackler Faculty of Medicine, Tel Aviv University, Tel Aviv, Israel

## Abstract

In breast cancer patients, the lungs are among the first sites of cancer metastasis, and in nearly one quarter of metastatic patients, the exclusive first event. Two common mouse models mimic breast cancer lung colonization and distal metastasis: an orthotopic model and intravenous (IV) cell injections. Gene expression analysis of pulmonary lesions from these two methods demonstrated high inter-model resemblance. However, microRNA (miRNA) expression profiles were not compared. In this study, we compared the overall miRNA expression profiles (miRNome) of the orthotopic and IV breast cancer metastasis models and identified significant miRNome changes between the two models. Overexpression of the most significant candidate, miR-96 or downregulation of its validated gene-target, ABCE1 reduced cancer cells 2D/3D cell movement and proliferation in vitro, and abated tumor growth and metastasis formation in vivo. Human data analysis further strengthened miR-96/ABCE1 role in breast cancer tumor aggression. Taken together, our results indicate that IV- and orthotopic models differ by their miRNome. Specifically in our study, breast cancer aggressiveness was dictated by miR-96 regulating ABCE1. Overall, miRNome analysis of various metastatic cancer models may lead to the identification of candidate genes critical to metastasis development.

## Background

Breast cancer is the second most commonly diagnosed cancer after skin cancer, and the second leading cause of cancer deaths among women after lung cancer^1–3^. Tumor metastasis—the migration of tumor cells from a primary site to progressively colonize distant organs-is a major cause of cancer-related deaths. However, only a unique subpopulation of primary tumor cells that acquire special genetic and epigenetic changes are able to successfully metastasize. Despite the inefficiency of metastatic formation (animal models estimate that only ~0.02% of tumor cells develop metastatic abilities^4^), most breast cancer complications are due to metastatic development in regional lymph nodes and distal organs^5^. The lungs are amongst the first-and often the exclusive first-sites of metastasis in nearly one quarter of metastatic breast cancer patients^6^. The metastatic process is mediated by complex crosstalk between tumor cells and their supporting stroma^7^ and cannot be fully mimicked in vitro. Hence, research on breast cancer metastasis formation has greatly benefited from the use of mouse models^8^. In such models, recapitulation of the stages of tumor progression enables analysis of the underlying molecular mechanisms of breast cancer progression and metastasis. Orthotopic models, in which cancer cells are introduced directly into the mammary fat pad, share many features of human primary tumor growth and metastasis. However, the orthotopic model is not well suited for breast cancer subtypes that metastasize slowly. A common murine model for the study of later stages of the metastatic cascade entails systemic injection of cancer cells, usually intravenously (IV). Injection of breast cancer cells directly into the venous system results in high rates of lung seeding and lower rates of liver/brain/bone seeding, effectively mimicking metastatic growth in these organs^9,10^. Advocates of the IV model argue that it is a quick and easy way to emulate metastatic lung lesions, especially with cell lines that typically require long periods of time to metastasize. However, others argue that this model does not adequately mimic human metastatic breast cancer because systemically-introduced cancer cells recapitulate only metastatic colonization and circumvent the primary disease^11^. Furthermore, systemic metastasis models do not undergo the same mutations as primary tumor cells that produce distant metastases^12^. Nonetheless, gene expression profiling of pulmonary lesions from orthotopic and IV models demonstrates high inter-model resemblance^13^ and justifies the continuation of the latter model for lung metastasis research.

MicroRNAs (miRNAs) are non-coding small RNAs (~22 nt) that negatively regulate gene expression and that are highly associated with tumorigenicity, invasion, and metastasis^14^. Each miRNA can regulate multiple genes that act in concert on the same biological pathway and that considerably influence its function^15^. In breast cancer, miRNAs have been shown to regulate self-renewal and differentiation of cancer stem cells; control epithelial to mesenchymal transition (EMT); and suppress cancer cell migration and invasion in vitro, and distal pulmonary metastasis in vivo^16^. Despite evidence of the importance of gene regulation by miRNAs, the typical magnitude of observed mRNA repression by miRNAs is relatively small^17–19^. This discrepancy between minor mRNA expression changes and significant phenotypic effect could be due to the reported role of miRNA in direct translational repression of hundreds of genes, in addition to its role on mRNA stability; miRNA has been shown to modulate ribosome initiation, elongation, and termination, thereby affecting mRNA translation independent of mRNA expression^20^.

In this study, we assessed differences in overall miRNA expression profiles (miRNome) between the orthotopic and IV breast cancer metastasis models, and identified miR-96 as an active suppressor of the metastatic process. We identified ABCE1 as a direct target of miR-96 and validated the suppressive effect of miR-96 and ABCE1 on breast cancer cell migration, invasion, and proliferation. We then demonstrated their role in reducing tumor growth and metastatic activity in vivo. Finally, by comparing human clinical data and survival with tumor RNA expression, we showed that miR-96 and ABCE1 have a significant role in breast cancer progression.

## Materials and methods

### Development of orthotopic and IV breast cancer mouse models

Six-week-old female BALB/c mice were purchased from Envigo RMS Laboratories (Ness Ziona, Israel). Mice were maintained under the guidelines of the University of Tel Aviv Institutional Animal Care and Use Committee. Orthotopic tumors were induced by exposing the fourth (inguinal) mammary fat pad and injecting it with 2 × 10^5^ 4T1 cells suspended in 50 μL of PBS (Biological Industries). Tumor growth was assessed by measuring individual tumors with calipers and calculating tumor volume: Tumor volume (mm^3^) = (width × length^2^)/2. The IV metastatic breast model was achieved by tail vein injection of 10^4^ 4T1 cells suspended in 100 μL of PBS (Biological Industries).

### NanoString miRNA expression profiling

The multiplexed NanoString nCounter Mouse v1.5 miRNA Expression Assay (NanoString Technologies) was used to profile 581 mouse miRNAs. The assay was performed as previously described^21^. The mean value of negative controls was set as the lower threshold for each sample and microRNAs with at least 50% of their values equal to or lower than the lower threshold were excluded. Normalization of raw data and differential expression analysis was conducted with the DEseq2 package^22^ and in-house script under R software.

### Cell lines and expression regulation

Breast cancer cell lines (4T1, MDA-231, and HS578), HeLa cells, and HEK-293T cells were described previously^23^. Cells were cultured in Dulbecco’s Modified Eagle’s Medium (Biological Industries) supplemented with 10% fetal bovine serum (GIBCO). Before use, each cell line was confirmed to have no mycoplasma contamination using the EZ-PCR Mycoplasma Test Kit (Biological Industries). Transient and stable cell lines expressing scrambled control miRNA or miRNA-96 overexpression were established as described previously^24^. Construction of ABCE1 cDNA plasmid and stable transfection was established as described previously^25^. Short hairpin RNAs (shRNAs) for ABCE1 and scrambled vector were established as described previously^26^. Plasmids pLKO.1-ABCE1 and pLKO.1-scrambled were purchased from Dharmacon. Short interfering RNAs (siRNAs) against ABCE1 and scrambled (control) were purchased from Integrated DNA Technologies.

### RNA analysis

Total RNA extraction and reverse transcription were performed as previously described^27^. mRNA was reverse transcribed with random primers and SuperScriptIII reverse transcriptase (Thermo Fisher). Reverse transcription for specific miRNAs was performed with TaqMan miRNA Assays (Thermo Fisher). Single miRNA and mRNA expression was tested similarly using TaqMan Universal PCR Master Mix (No AmpErase UNG; Thermo Fisher) and SYBR green PCR master mix (Thermo Fisher), respectively, by means of the StepOnePlus real-time PCR system (Thermo Fisher). Specific primer pairs for mRNA expression detection were ordered from IDT (supplementary table 1). Expression values were calculated based on the comparative threshold cycle method. miRNA levels were normalized to U6 snRNA, and mRNA expression levels were normalized to human GAPDH or mouse actin. RNA-seq libraries were constructed using the TruSeq Stranded Total RNA Library Prep Kit (Illumina), and sequencing was performed on a HiSeq 2500 (Illumina), with 100 bp paired-end reads. Reads were mapped to the *Mus Musculus* reference genome GRCm38 using STAR v2.4.2a and annotated with Ensembl release 82. Expression levels for each gene were quantified using HTseq-count^28^. Differential expression analysis was performed using DESeq2^22^. mRNAs with count <30 in all samples were excluded. Gene transcripts displaying absolute fold-change ≥1.2 and adjusted *p*-value < 0.05 were considered differentially expressed.

### IncuCyte live cell imaging system

The IncuCyte S3 system and software (Sartorius) were used for migration and invasion assays according to the manufacturer’s protocols. In 96-well plates (IncuCyte ImageLock Plates 4379), 2 × 10^4^ 4T1 or 3 × 10^4^ HS578 cells/well were seeded for each assay. Images were analyzed with the IncuCyte HD software (Sartorius) and the results presented as relative wound densities and standard deviations for each time point. Relative wound density (%) represents the cell density in the scratch area relative to that outside the scratch, as a function of elapsed time. Experiments were performed in replicates of five per condition.

### Colony formation assay

HS578 and 4T1 cells (scrambled/miR-96 OE/Abce1 KD/miR-96 OE + Abce1 OE) were seeded in 6-well plates (1 × 10^3^ cells per plate) and cultured for 8 days (4T1) or 14 days (HS578). The colonies were stained with 0.5% crystal violet and for 30 min after fixation with 10% methanol for 15 min. Quantification of stained, fixed colonies was done using ImageJ software (NIH). All experiments were performed in triplicates.

### Anchorage-independent growth ability assay

1 × 10^3^ 4T1 and HS578 cells (scrambled/miR-96 OE/Abce1 KD/miR-96 OE + Abce1 OE) were detached by Trypsin and suspended in 2 mL complete medium plus 0.33% noble agar (Invitrogen). The mixture was seeded in a six-well plate containing 0.66% complete medium/noble agar mixture. Colony sizes were measured with an ocular micrometer after 13 days (4T1) or 23 days (HS578) incubation and colonies greater than 0.1 mm in diameter were scored. All experiments were performed in triplicates.

### Dual luciferase reporter assay

The predicted binding site for miR-96 on the 3’UTR of ABCE1 was PCR-amplified as previously described^29^ and cloned into the psiCHECK-2 plasmid (Promega). Negative control of ABCE1 was achieved by substituting 3 nucleotides in the seed binding region of the cloned 3’UTRs using the QuikChange Lightning SDM kit (Agilent). HEK-293T and HeLa cells were seeded in 24-well plates supplemented with 10% FBS (GIBCO). Cells were transfected using Lipofectamine 2000 (Rhenium), 5 ng of the psiCHECK-2 relevant clone, 10 ng of pEGFP, and 485 ng miRVec containing the desired pre-miRNA. Twenty-four hours following transfection, lysates were extracted and firefly and Renilla luciferase activities were measured using the Dual-Luciferase Reporter Assay System Kit (Promega). The Renilla luciferase results were normalized to the values of the firefly luciferase.

### Western blot analysis

Cells were homogenized with lysis buffer containing 50 mM Tris HCl (pH 7.6), 20 mM MgCl_2_, 150 mM NaCl, 0.5% NP40, and 5 units/mL Aprotinin (Sigma-Aldrich). Lysates were collected after centrifugation and protein concentrations determined using the Bio-Rad protein assay (Bio-Rad Laboratories). Lysates were resolved by SDS–polyacrylamide gel electrophoresis (SDS-PAGE) using 4–12% gels (Gentaur), and electrophoretically transferred to a nitrocellulose membrane. Membranes were blocked for 1–2 h in TBST buffer (0.02 M Tris HCl pH 7.5, 0.15 M NaCl, and 0.05% Tween 20) containing 5% milk, and then incubated with dilute primary antibody (supplementary table 2) in blocking solution overnight at 4 °C. Membranes were washed in TBST buffer 3 times, and incubated with a secondary antibody (supplementary table 3) for 45 min at room temperature. Immunoreactive bands were detected with enhanced chemiluminescence reagent (Thermo Fisher) and quantified using ImageJ software (NIH).

### Spheroid invasion assay

Spheroids were produced by the hanging-drop technique as previously described^30^. In brief, single-cell suspensions of 50,000 4T1 and HS578 cells/ml (scrambled/miR-96 OE/ABCE1 KD/miR-96 OE + ABCE1 OE) were prepared, and 10ul droplets were pipetted onto the inner side of a 10 cm petri dish lid. Dishes were filled with 5 mL of sterile PBS. The hanging drop cultures were incubated at 37 °C for 72 h (4T1) and 96 h (HS578) to generate spheroids. Spheroids were then collected, mixed with Matrigel (BD bioscience) and plated in 24-well plates. Photographs were taken using an inverted microscope at 0, 24, and 48 h after plating, and quantified using ImageJ software (NIH).

### miRNA target prioritization

Ten miRNA-target prediction programs (DIANA-microT^31^, miRanda^32^, miRTarget^33^, miRmap^34^, miRNAMap^35^, PicTar2^36^, PITA^37^, RNA22v2^38^, RNAhybrid^39^, and Targetscan^40^) were used to predict gene targets of miR-96. Targets predicted by fewer than eight prediction tools that were to be regulated by miR-96 were removed from downstream analysis. Next, pubmed.mineR^41^ was utilized to obtain and quantify the number of publication abstracts related to “cancer” for each miR-96 predicted gene target. Gene targets with <5 hits were filtered out from further analysis. Subsequently, staining profiles in human breast tumor tissue based on immunohistochemistry (IHC) were obtained from The Human Protein Atlas^42^ and were used for the assessment of IHC staining profiles of candidate targets that appeared in both the cancer related-miR-96 predicted target list and the RNA-seq differentially expressed transcript list. Candidate targets that presented a low or undetected staining level in more than 50% of breast tumors were removed.

### Human breast cancer data analysis

miR-96 and ABCE1 survival analysis was performed using the web tool miRpower^43^. miR-96 and *ABCE1* mRNA expression profiles from the Molecular Taxonomy of Breast Cancer International Consortium (METABRIC) database^44^ were obtained using the cBioPortal for Cancer Genomics (http://cbioportal.org)^45^. Analysis of variance (ANOVA) and post-hoc analyses were used to evaluate associations of miR-96 and *ABCE1* expression with tumor grade, tumor intrinsic subtype (based on PAM50 classification), and cancer stage.

## Results

### miRNome changes in IV- and orthotopic-derived lung metastases

**Fig. 1:**
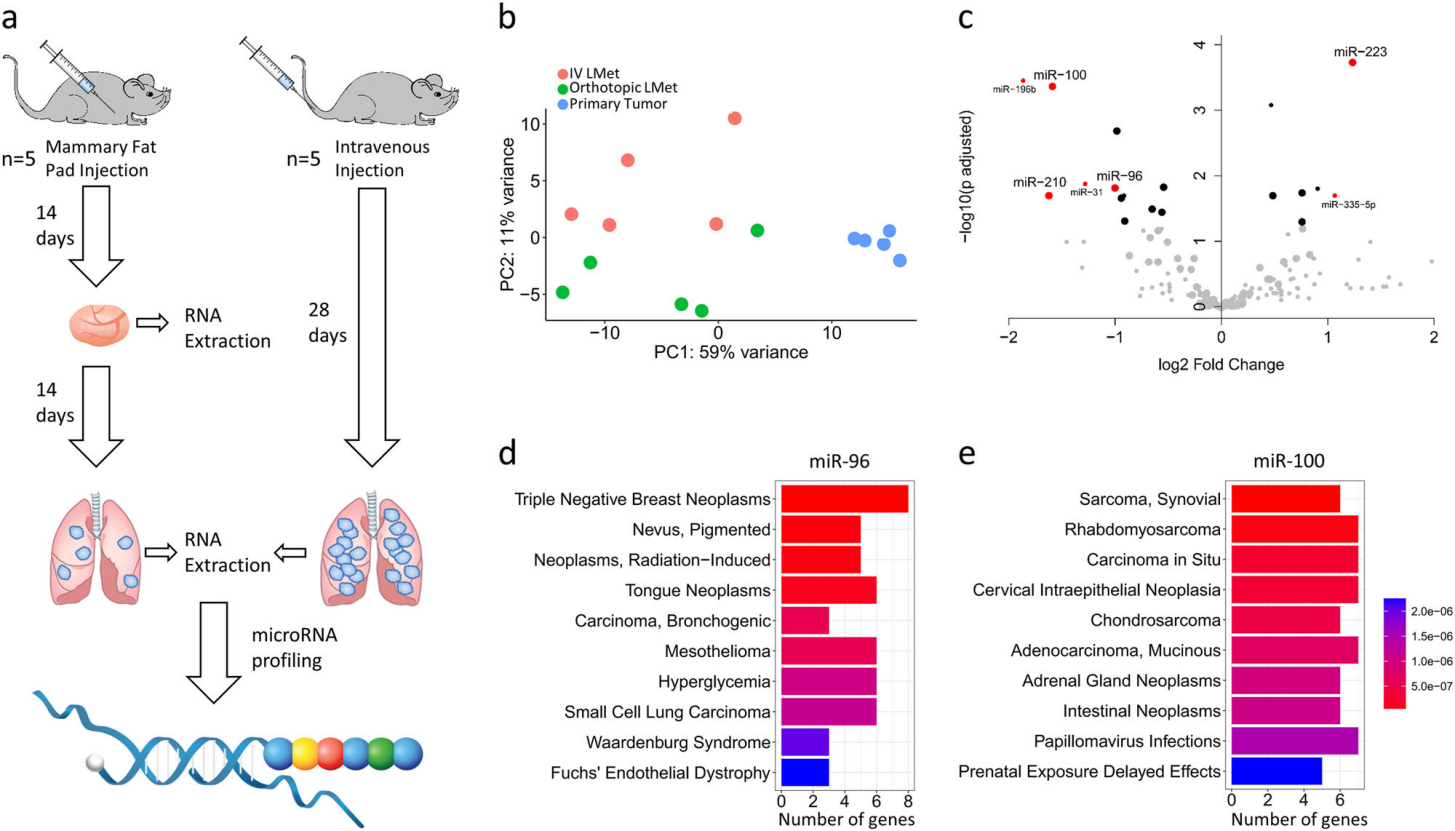
miRNome analysis of LMets from orthotopic and IV models reveals miR-96 as a potential metastasis suppressor gene. **a** Schematic representation of experimental design. Mice were injected with 4T1 cells orthotopically or intravenously. On day 14, primary tumors were excised from the orthotopic group, and RNA purified. On day 28, lungs were excised from mice in both groups, and 1-2 lung macrometastases isolated for RNA extraction and miRNA profiling. **b** PCA analysis of NanoString miRNA expression in primary tumors (blue), orthotopic LMets (green), and IV LMets (red) shows distinct characterization of each group. The primary tumor samples cluster very tightly compared to the diffuse clustering of the LMet samples. This indicates that as metastatic disease progresses, miRNA profiles become highly variable compared to those of the primary tumor. **c**. Filtration of significant miRNAs from miRNome analysis is presented as a volcano plot. Each dot represents a different miRNA: large dots represent miRNAs in the top quantile of expression, black dots represent miRNAs with adjusted *p*-value < 0.05, and red dots represent miRNAs with both a fold change >2 and adjusted *p*-value < 0.05. MeSH enrichment analysis for **d** Twenty-one miR-96 and **e** Twenty miR-100 validated gene-targets obtained from miRTarBase^46^. miR-96 enrichment results in breast cancer as a top enriched disease. The *X*-axis represents the number of genes reported to be involved in the diseases labeled in the *Y*-axis, colors represent adjusted *p-*values. Barcoded DNA and probe image in (a) was adapted from the NanoString website (https://www.nanostring.com/products/mirna-assays)

To assess differences in the miRNA expression profiles of the IV and orthotopic breast cancer models, 4T1 cells were injected into five BALB/c mice, either directly into the mammary fat pad or intravenously. On day 28, lung CT showed numerous lung metastases (LMets) in the IV group and 1–2 LMets in the orthotopic group. RNA was purified from primary tumors and macrometastases (two macrometastases were taken from each mouse, with the exception of one mouse from the orthotopic group that developed only one macrometastasis), followed by NanoString multiplex miRNA reading (Fig. 1a). Principal component analysis (PCA) revealed differential miRNA characterization of primary tumors, orthotopic LMets, and IV LMets (Fig. 1b). This suggests that miRNA expression profiles differ according to disease progression. To identify miRNAs that are key players in the early stages of metastatic development, we filtered the NanoString miRNA-expression data for miRNAs that matched three criteria: highly expressed in at least one of the LMet groups, with an adjusted *p*-value < 0.05, and expression fold change of >2. Combining these three filtration criteria resulted in four candidate miRNAs to be included in downstream analyses: miR-96, miR-100, miR-223, and miR-210 (Fig. 1c and supplementary table 4). We repeated the experiment (*n* = 3 in each group), using qPCR on RNA extracted from two LMets from each mouse to assess the expression of miR-96, miR-100, miR-223, and miR-210, and found that miR-100 and miR-96 displayed the same differential expression patterns (IV > orthotopic, *p* < 0.05), while miR-210 and miR-223 did not (supplementary figure 1). Next, we downloaded curated gene candidates of miR-96 and miR-100 from miRTarBase^46^ and tested the Medical Subject Headings (MeSH) enrichment for each gene set using Clusterprofiler^47^. The top enriched value for miR-96 was triple negative breast cancer while miR-100 gene targets did not demonstrate enrichment for breast cancer (Fig. 1d, e). We thus decided to focus on the most significant candidate, miR-96, and its effect on the metastatic process.

### miR-96 directly targets ABCE1 and downregulates its expression

Orthotopic-derived LMets had lower miR-96 expression than LMets of the IV model. We hypothesized that miR-96 upregulation may preclude breast cancer primary tumor cells from escaping the primary tumor environment. Alternatively, primary tumor cells that downregulate miR-96 expression may disseminate more easily. To identify the gene that is most affected by miR-96 via miRNA-gene targeting, we stably overexpressed miR-96 or scrambled vector in 4T1 murine breast carcinoma cell line and orthotopically injected the resulting cells into BALB/c female mice. Fourteen days after cell injection, tumors were removed and RNA was extracted from each tumor and sent for RNA-seq. We performed differential expression analysis and focused on genes downregulated in miR-96 OE tumors compared to the scrambled control group (assuming reciprocal regulation of miRNA-mRNA). Selecting transcripts that were expressed above the global median expression, set as the expression level cutoff, and using an adjusted *p*-value < 0.05, we identified 127 differentially expressed transcripts (supplementary table 4). Using miRNA-mRNA target prediction tools and curation for cancer-gene connection (PubMed), we assembled a list of cancer related-miR-96 predicted targets (944 genes, supplementary table 8). The overlap of this list is presented with the RNA-seq differentially expressed transcripts (127 genes, supplementary table 4) reduced the number of candidates to twelve (*Abce1, Lcp1, Ovol1, Sgk3, Spata13, Ivns1abp, Clptm1l*, *Camta1, Pmepa1, Rdh11, Prkci* and *Hmgcr*). Utilizing the Human Protein Atlas^42^, we noted that ABCE1 was the only candidate with moderate to strong IHC expression profile in breast cancer tissues (supplementary table 9). We therefore continued to explore its role in breast cancer metastasis formation.

**Fig. 2:**
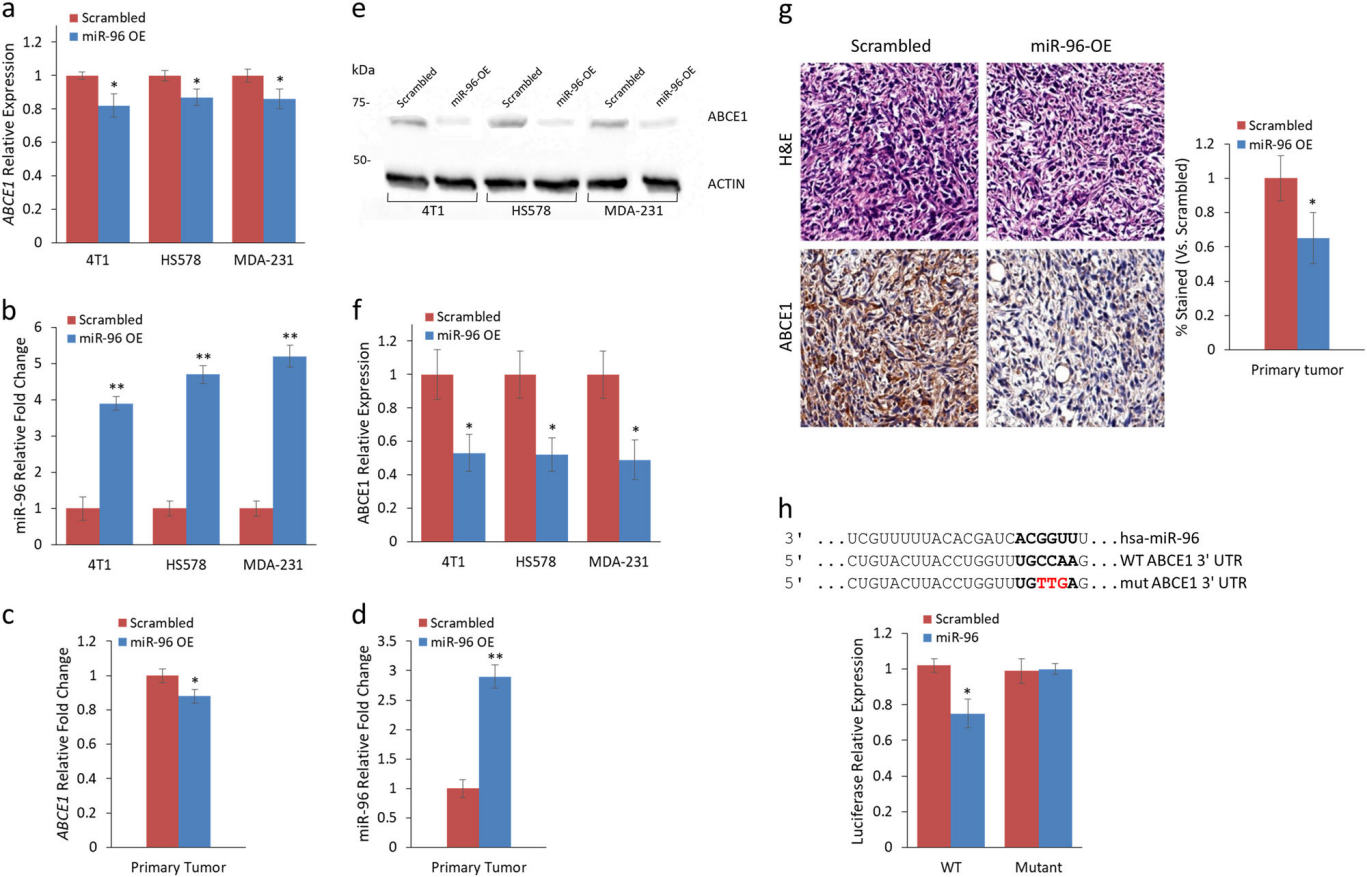
ABCE1 gene and protein expression are reduced in direct response to miR-96 overexpression in vitro and in vivo. **a** *ABCE1* and **b** miR-96 expression levels in 4T1, HS578, and MDA-231 cell lines expressing miR-96 OE or scrambled control. *ABCE1* expression is reduced in all cell lines with miR-96 OE. **c** *ABCE1* and **d** miR-96 expression in primary tumors of mice injected with miR-96 overexpressing or scrambled control 4T1 cells. *ABCE1* and miR-96 expression levels are inversely correlated. **e** Western blot of ABCE1 in breast cancer cell lines and **f** calculated ABCE1 protein expression in cell lines with miR-96 OE or scrambled control. ABCE1 expression is twofold decreased in miR-96 OE cells compared to scrambled control. **g** H&E and immunohistochemistry for Abce1 of resected murine primary breast tumors. Reduced Abce1 staining is seen in miR-96 OE compared to Scrambled. **h** Predicted binding site (indicated by bold letters) for hsa-miR-96 on the ABCE1 3′-UTR and Luciferase activity 24 h following co-transfection of HeLa cells with hsa-miR-96 and ABCE1 WT or Mut 3′-UTR construct. Wild type (WT) and mutant (Mut) miR-96 binding sites are presented. Red nucleotides represent the three mutated nucleotides in the miR-96 seed binding site. Data are presented as mean+/− SEM. *p < 0.05, **p < 0.01

To evaluate ABCE1 as a miR-96 target in vitro and in vivo, we examined its expression in 4T1, MDA-231, and HS578 breast cancer cells that overexpress miR-96 or scrambled miRNA. *ABCE1* RNA expression in miR-96 OE cells was reduced across all cell lines (Fig. 2a). miR-96 expression was quantified to validate its reciprocal expression relative to *ABCE1* (Fig. 2b). Next, we assessed *Abce1* and miR-96 expression in mouse primary tumors and noted a significant mean decrease of 10–15% in *Abce1* expression (Fig. 2c) and 3.8-fold increase in miR-96 expression (Fig. 2d) when comparing miR-96 OE vs. scrambled expression level. Western blot analysis of ABCE1 in 4T1, HS578, and MDA-231 revealed a reduction of more than 50% in ABCE1 protein expression in miR-96 OE compared to scrambled cells (Fig. 2e, f). Immunohistochemical analysis further corroborated this trend, with extensive reduction of ABCE1 expression in miR-96 OE primary tumors and IV LMets but not in orthotopic LMets (Fig. 2g). To assess whether miR-96 directly regulates ABCE1, we conducted luciferase reporter assays on HEK293 and HeLa cells co-transfected with miR-96, and WT or mutant *ABCE1* (Fig. 2h). A significant decrease in luciferase activity was observed in cells that received WT *ABCE1* compared with cells of the mutated construct, indicating that miR-96 directly regulates *ABCE1* expression.

### miR-96 overexpression and ABCE1 knockdown reduce 2D and 3D migration, invasion, and proliferation of breast cancer cells

**Fig. 3:**
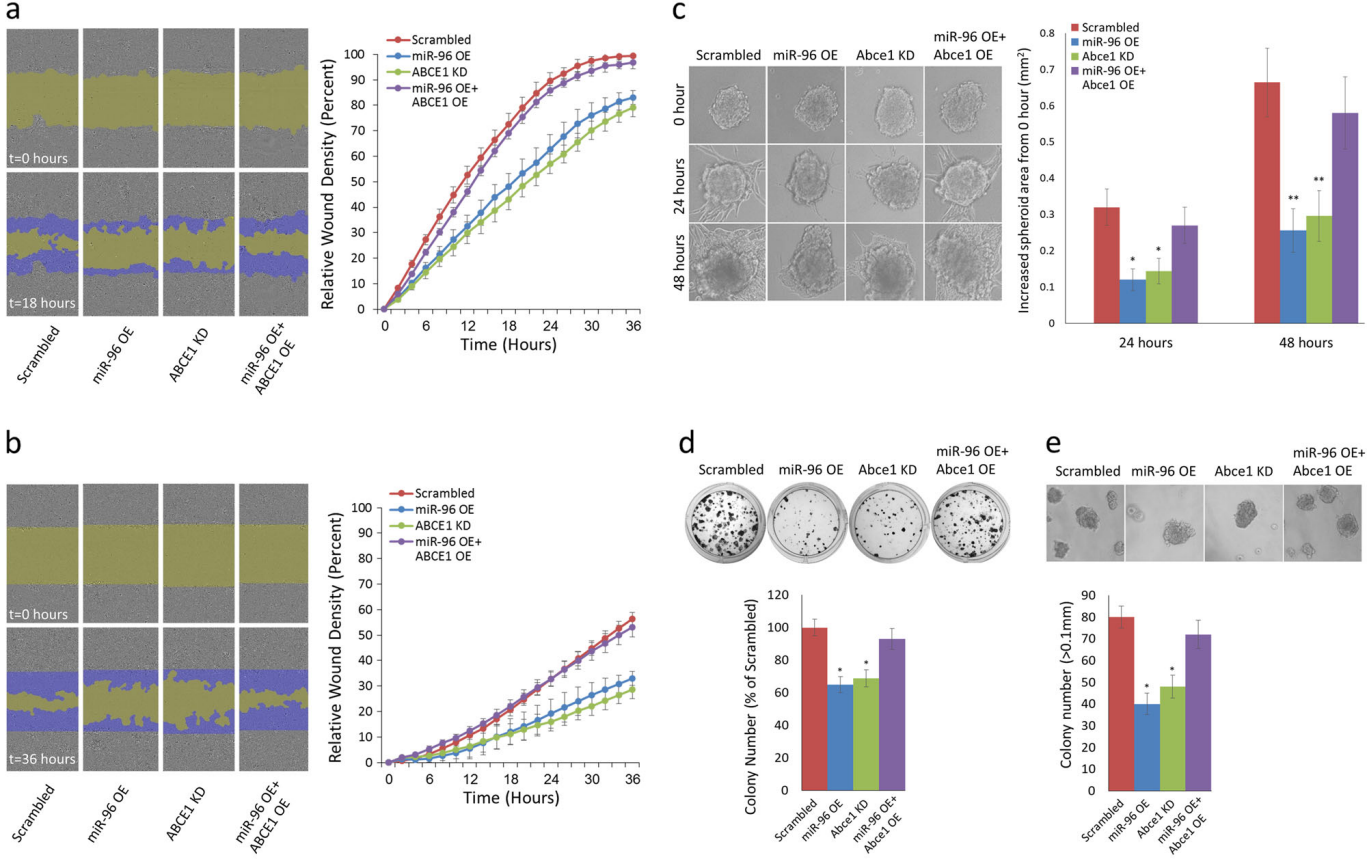
miR-96 OE and ABCE1 KD downregulates 2D and 3D migration, invasion, and proliferation of breast cancer cells. **a** Representative images of a migration assay (left) and mean scratch area closure over time (right). The upper row represents time 0 and the bottom row represents 18 h post-scratch. HS578 cells (scrambled/miR-96 OE/ABCE1 KD/miR-96 OE + ABCE1 OE) are shown in gray, the green area represents the scratch, and migrating cells are indicated in dark blue. The migration assay mean scratch area closure over time reveals a delay in the rate of migration of cells that overexpress miR-96 or underexpress ABCE1 compared to the scrambled control. Overexpression of ABCE1 in miR-96 OE cells abrogates miR-96 migration inhibition. Error bars were calculated for each measurement. **b** Representative images of an invasion assay (left) and mean scratch area closure over time (right). The upper image represents time 0 and the bottom image represents 36 h post-scratch. Invading HS578 cells (scrambled/miR-96 OE/ABCE1 KD/miR-96 OE + ABCE1 OE) are indicated in dark blue. The invasion assay mean scratch area closure over time demonstrates slower invasion rates for miR-96 OE and ABCE1 KD cells. Overexpression of ABCE1 in miR-96 OE cells reversed miR-96 inhibitory effect on invasion. Error bars were calculated for each measurement. **c** Representative images from 4T1 spheroid invasion assays on the day of seeding (0 h), 24 h, and 48 h after seeding (left) and quantification of results (right). A significant decrease of spheroid invasion was noted for miR-96 OE and Abce1 KD cells. Spheroids co-overexpressing miR-96 and Abce1 invaded similarly to scrambled control spheroids. **d** Representative images (top) and quantification (bottom) from colony formation assays. **e** Representative images (top) and quantification (bottom) of colony numbers from anchorage-independent growth assay. Only colonies larger than 0.1 mm in diameter were counted. Data are presented as mean +/− SEM. *p < 0.05, **p < 0.01

To examine the effect of miR-96 OE and its predicted downregulation of ABCE1 on cell migration, we conducted a scratch wound migration assay using the IncuCyte Live Cell Imaging System (Sartorius) on HS578 and 4T1 breast carcinoma cells that stably overexpress miR-96, underexpress ABCE1, or overexpress both miR-96 and ABCE1. A significant reduction in cell migration was observed in miR-96 OE and ABCE1 knockdown (KD) cells compared to the scrambled control (Fig. 3a). Interestingly, overexpressing ABCE1 in miR-96 OE cells abrogated the inhibitory effect on cell migration. We then used the IncuCyte system to conduct scratch wound invasion assays on these cells, and noted a similar trend of reduced scratch closure in the miR-96 OE and ABCE1 KD cells compared to the scrambled control (Fig. 3b). As in the migration assay, ABCE1 OE reversed the inhibitory effect of miR-96 on cell invasion. We then assessed the effect of miR-96 OE and ABCE1 KD on cell migration within a native-like 3D tumor microenvironment using an extracellular matrix assay. Utilizing the hanging drop technique^30^, we developed tumor spheroids, which we embedded in Matrigel, and monitored for 48 h. miR-96 OE and Abce1 KD spheroids demonstrated significantly less invasion compared to Scrambled, while ABCE1 overexpression in miR-96 OE cells rescued invasion capacity (Fig. 3c). Colony formation (Fig. 3d) and anchorage-independent growth assays (Fig. 3e) demonstrated reduced activity for miR-96 OE and Abce1 KD, compared to Scrambled or miR-96 OE + ABCE1 OE cells. These results suggest that miR-96 functions as a tumor-suppressive miRNA in breast cancer by regulating ABCE1 expression.

### miR-96 OE and ABCE1 KD reduce breast tumor proliferation and lung metastases in vivo

**Fig. 4:**
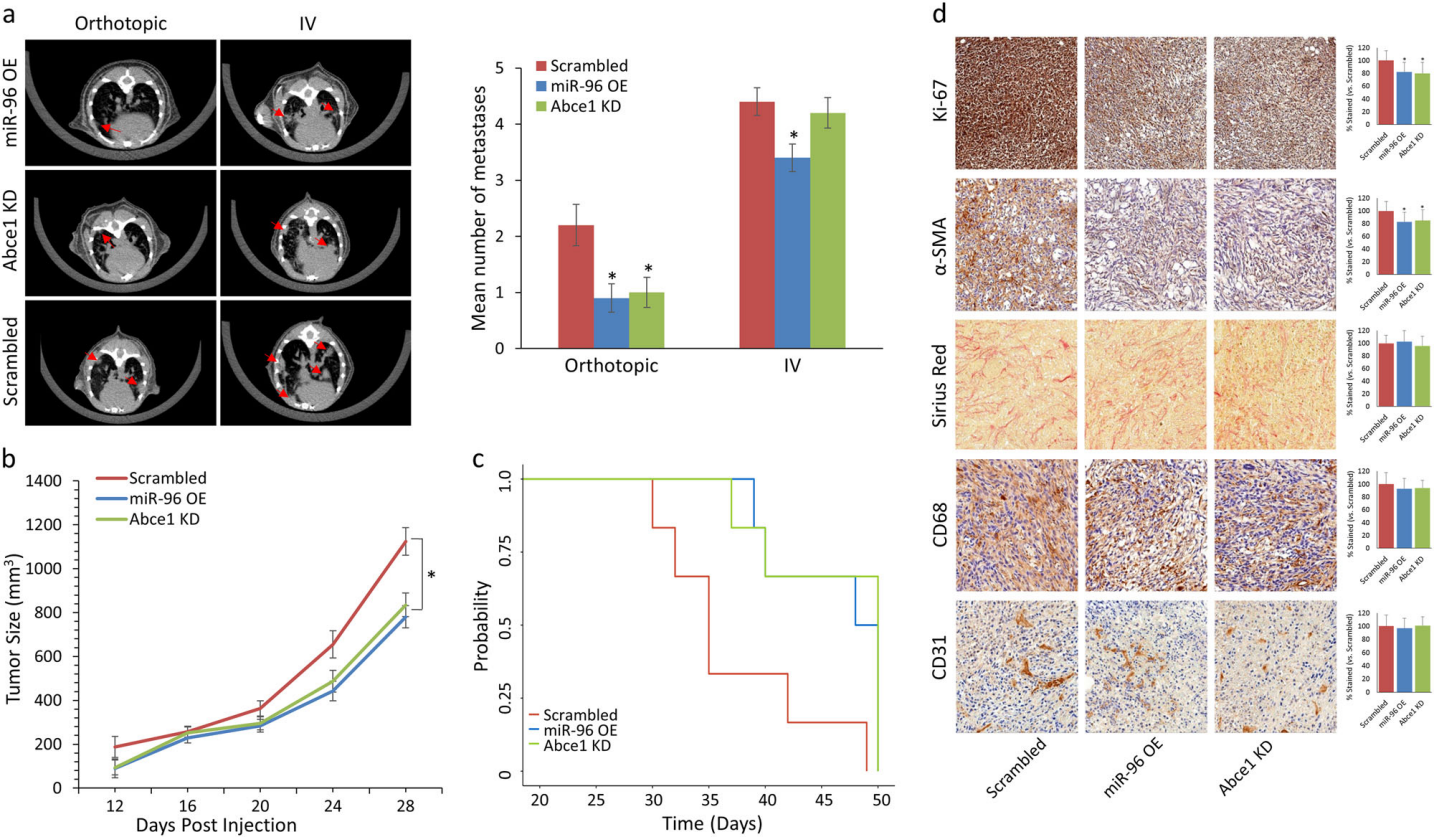
miR-96 OE and Abce1 KD reduce breast tumor proliferation and lung metastases in vivo. **a** Lung CT scans performed on day 28 (orthotopic) and day 21 (IV) post-4T1 injection (left) and quantification of LMets in microCT (right) show significantly fewer metastatic growths (indicated by red arrows) in mice that orthotopically received miR-96 OE or Abce1 KD cells compared to the scrambled control. IV injection of miR-96 OE cells resulted in fewer lung foci compared to Scrambled. **b** Primary tumor volume analysis showed reduced tumor volume of miR-96 OE and Abce1 KD groups compared to Scrambled **c** Kaplan-Meier survival analysis demonstrated increased overall survival of mice that received miR-96 OE or Abce1 KD cells compared to the scrambled control (*p* = 0.06). **d** Representative images of Ki-67, α-SMA, Sirius Red, CD68, and CD31 staining in resected tumors from Scrambled, miR-96 OE, and Abce1 KD groups as indicated. At least 30 fields were analyzed from each group. *n* = 3 for each group. Scale bar = 100 μm. Magnification ×20. Quantifications of staining are presented as percent relative to control. miR-96 OE and Abce1 KD tumors demonstrated decreased proliferation (Ki-67) and were associated with significant reduction in activated αSMA + cancer associated fibroblasts in the tumor microenvironment, while collagen deposition (Sirius red staining) was not altered. Data are presented as mean +/− SEM. *p < 0.05

Intrigued by our in vitro results, we set out to analyze the functional roles of miR-96 and ABCE1 in tumor growth and lung metastases formation in vivo. To this end, miR-96 OE, Abce1 KD cells, or scrambled 4T1 cells were introduced orthotopically or intravenously into BALB/c female mice. Lung CT scans revealed a significant reduction in orthotopic LMets in the miR-96 OE and Abce1 KD compared to the scrambled group (Fig. 4a). miR-96 OE cells resulted in fewer lung foci when introduced intravenously compared to Scrambled or Abce1 KD cells (Fig. 4a). Remarkably, overexpressing miR-96 or knocking down Abce1 expression significantly inhibited tumor growth and improved overall survival, suggesting that miR-96 regulates cancer progression by targeting Abce1 (Fig. 4b, c). Since cancer cells may modulate the microenvironment using intercellular communication, we evaluated stromal changes by conducting immunohistochemistry (IHC) staining of tumor tissues derived from miR-96 OE, Abce1 KD, and scrambled control cells. IHC analysis showed that miR-96 OE and Abce1 KD tumors exhibited lower proliferation (Ki67 staining) compared to Scrambled (Fig. 4d). Analysis of fibroblast activation (αSMA staining) indicated that overexpressing miR-96 or knockdown of Abce1 in tumor cells was associated with significant reduction in activated αSMA in the tumor stroma, while collagen deposition (Sirius red staining) was not significantly altered (Fig. 4d). Macrophage infiltration (CD68 staining) and angiogenesis (CD31 staining) were also insignificantly affected in miR-96 OE and Abce1 KD derived tumors compared to scrambled (Fig. 4d). These results indicate that reduced Abce1 expression, induced by miR-96 OE, is functionally important for facilitating tumor growth and metastases formation, as well as fibroblast activation in the tumor microenvironment.

### Clinical significance of miR-96 and *ABCE1* expression on survival of human patients

**Fig. 5:**
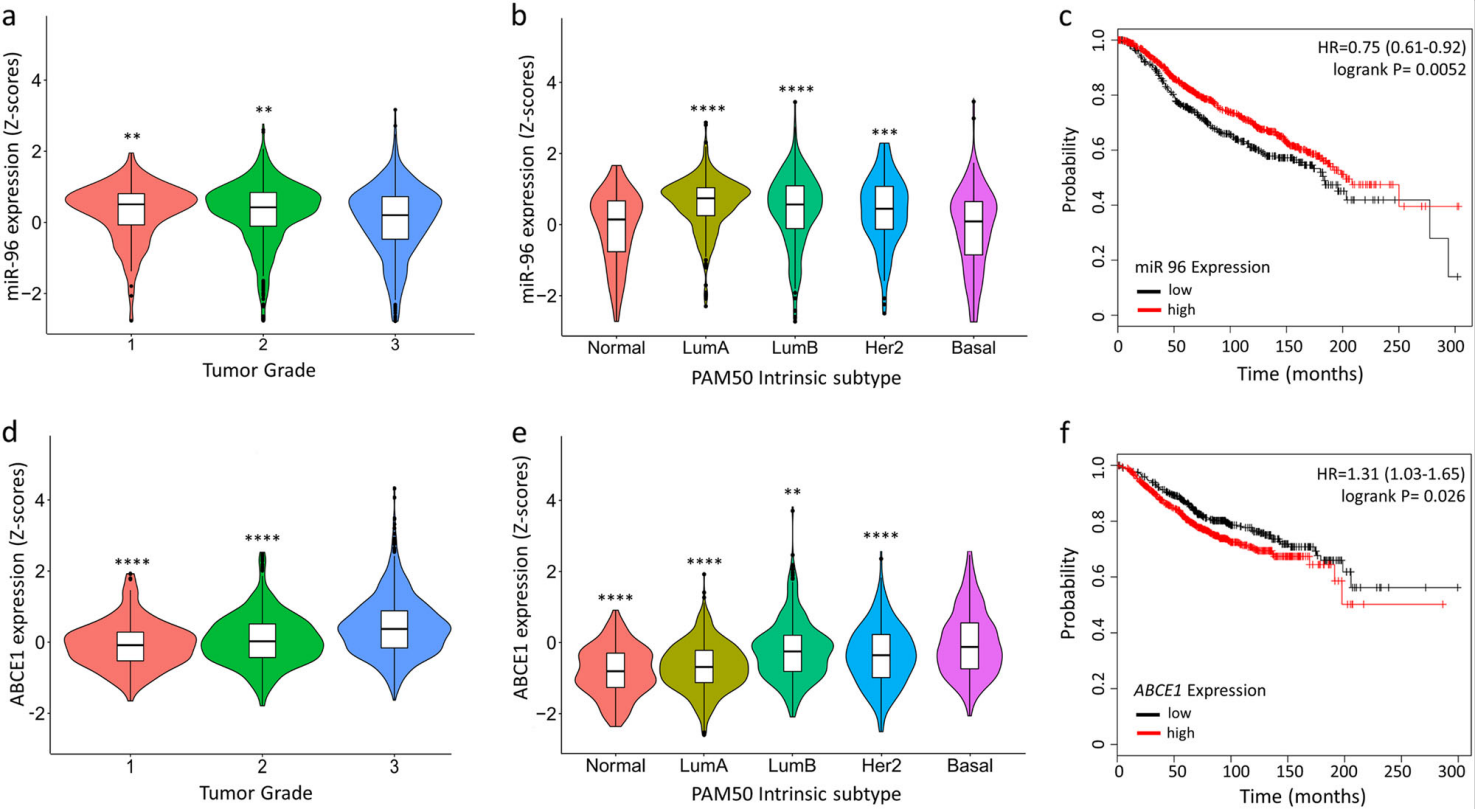
Increased miR-96 expression and reduced ABCE1 expression are correlated with lower tumor grade, preferable intrinsic tumor subtype, and longer survival in humans. Clinical data of 1,262 breast cancer patients, of which expression levels of miR-96 and *ABCE1* were available, were obtained from the METABRIC database. **a** miR-96 expression was inversely correlated with tumor grade and **b** basal type breast cancer. **c** Kaplan–Meier survival analysis demonstrates longer overall survival of patients with breast tumors that highly express miR-96. **d** *ABCE1* expression was correlated with higher tumor grade and **e** basal type breast cancer. **f** Kaplan–Meier survival analysis demonstrates longer overall survival of patients with breast tumors that lowly express *ABCE1*. Data are presented as mean +/- SEM. **p < 0.01, ***p < 0.001, ****p < 0.0001

To assess the relevance of miR-96 and *ABCE1* expression level in human breast cancer, we referred to the METABRIC^44^ database, which contains information from 1,262 breast cancer patients, whose miR-96 and *ABCE1* expression levels and clinical attributes (tumor grade, PAM50 intrinsic subtype, tumor stage, and survival status) are available. Using these data, we found that miR-96 expression was inversely correlated with basal intrinsic subtype (Fig. 5a) and higher tumor grade (Fig. 5b) while *ABCE1* expression was correlated with basal intrinsic subtype (Fig. 5d) and higher tumor grade (Fig. 5e). Increased survival was reported for patients with tumors that highly expressed miR-96 (Fig. 3c), as well as those with low *ABCE1* expression (Fig. 3f). Taken together, these data implicate an inverse relationship between the oncosupressive role of miR-96 and its oncogenic target, ABCE1, in breast cancer tumor aggression and survival.

## Discussion

As a means of exploring the underlying mechanisms of cancer biology, as well as developing better clinical models to test novel therapeutic strategies, mouse models provide critical insights into breast cancer research. Approximately 90% of breast cancer-related mortality results from the development metastatic disease^48^, thus many studies explore only the advanced stages of cancer, often using cell line-derived models^11^. Orthotopic metastasis models, in which the natural course of metastatic disease is recapitulated, requires weeks to months to generate metastatic growths. To bypass this long waiting period, many studies resort to IV models in which cancer cells are injected directly into the blood stream, essentially mimicking the advanced stages of the metastatic cascade. Genomic profile comparisons of lung metastases derived from orthotopic and IV breast cancer models were previously found to be indistinguishable^13^, confirming the IV model’s relevance in this field. In the current study, we analyzed the miRNome of breast cancer pulmonary metastatic growths in the murine orthotopic and IV models, and discovered that despite the reported similarity of mRNA profiles, these models differ by their miRNA signature. Additionally, by applying expression and functional enrichment analyses, we identified miR-96 as a critical regulator of breast cancer metastasis.

Papers describing the effect of miR-96 on breast carcinoma report inconsistent results, with an oncogenic role observed in some^49,50^, and a suppressive role in others^24,51^. The cellular and biological mechanisms of miR-96-mediated cancer cell regulation have been described in relation to a variety of processes, including cell motility (migration, invasion, and EMT)^50^, proliferation^52^, and angiogenesis^53^.

To better understand the mechanism by which miR-96 regulates the metastatic process, and to narrow down the effect to a specific gene, we used multiple approaches: RNA-seq analysis; miRNA-mRNA target prediction tools; curation for cancer related genes; and, protein expression data from the Human Protein Atlas^42^. We identified ABCE1 as the key target gene of miR-96. ABCE1 belongs to the family of ATP-binding cassette (ABC) transporter proteins, which are reported to be involved in translation, DNA repair, and chromosome maintenance. ABCE1 is highly expressed in breast cancer^54^ and has been found to directly affect cytoskeleton rearrangement^55^ by interacting with β-actin. Downregulation of ABCE1 was found to inhibit proliferation and invasion in breast cancer cells^56^.

In this study, we showed that ABCE1 is downregulated in human and mouse breast cancer cell lines, both on the RNA and protein levels, in response to miR-96 overexpression. Similar results were obtained in vivo, demonstrating a statistically significant mean decrease in ABCE1 RNA and protein expression in mouse primary tumors with miR-96 overexpression. Using a dual-Luciferase reporter assay, we confirmed the direct regulation of *ABCE1* transcription by miR-96.

We demonstrated that both overexpression of miR-96 and downregulation of ABCE1 result in significantly reduced breast cancer cell migration and invasion in 2D culture, and to even greater extent in 3D culture. Cells grown under classic 2D culture conditions behave differently from the same cell types grown in vivo; planar 2D growth geometrically constrains the cells, forcing an artificially imposed basal lateral attachment, resulting in genetic upregulation of cell cycling and metabolism as manifested through enhanced proliferation and extreme cell spreading^57^. To overcome the inherent limitations of 2D systems, 3D aggregates, known as multicellular tumor spheroids, are used to mimic important relational characteristics in vitro that are typically observed in tumors in vivo. In both 2D and 3D culture assays, we found that overexpressing ABCE1 in miR-96 OE cells overturned the oncosuppressive effect of miR-96, emphasizing its regulatory role in this complex process. Likewise, simultaneously inducing miR-96 expression and decreasing ABCE1 expression resulted in reduced anchorage dependent and independent growth, whereas overexpressing ABCE1 reversed this effect. We also demonstrated that miR-96 and ABCE1 play central roles in abating tumor growth and dissemination in vivo: either increasing miR-96 or decreasing ABCE1 in tumor cells was sufficient to significantly decrease tumor growth and metastasis formation.

The tumor growth-promoting functions of the miR-96 and ABCE1 could be partially mediated by the crosstalk between tumor cells and cancer associated fibroblasts (CAFs), which we found to be downregulated in miR-96 OE and Abce1 KD groups compared to Scrambled. CAFs are known to support tumorigenesis by stimulating cancer cell proliferation and invasion^58^. miR-96 expression has been shown to be significantly lower in CAFs compared to non-cancer fibroblasts^59^ while ABCE1 has been found to be overexpressed in the stromal compartment of cancer patients^60^. Our findings confirm these observations and indicate that in addition to its direct effect on tumor cells, the oncosuppressive effect of miR-96 may also be critical in the tumor microenvironment. Clinical and gene expression data from human breast cancer patients further corroborated the roles of miR-96 and ABCE1 in tumor aggressiveness.

## Conclusions

We demonstrated that IV- and orthotopic-derived breast cancer LMets differ in their miRNome. These findings imply that even though the IV model is a valid and important tool for studying lung colonization, significant differences in gene regulation mechanisms may exist between the two models. Analyzing those differences in various metastatic cancer models may lead to the identification of candidate genes critical to the metastatic process. Furthermore, using bioinformatics analysis, biological validation experiments, and human breast cancer data we identified miR-96 as an active metastasis suppressor gene and demonstrated that its ability to reduce metastatic activity depends on its regulation of ABCE1.

## Electronic supplementary material

Supplementary figure 1

Caption for supplementary figure 1

Supplementary table 1

Supplementary table 2

Supplementary table 3

Supplementary table 4

Supplementary table 5

Supplementary table 6

Supplementary table 7

Supplementary table 8

Supplementary table 9

## Data Availability

The datasets used and/or analyzed during the current study are available from the corresponding author on reasonable request.

## References

[1] DeSantis Carol E., Ma Jiemin, Goding Sauer Ann, Newman Lisa A., Jemal Ahmedin (2017). Breast cancer statistics, 2017, racial disparity in mortality by state. CA: A Cancer Journal for Clinicians.

[2] Siegel RL, Miller KD, Jemal A (2017). Cancer statistics, 2017. Cancer J. Clin..

[3] Ferlay J (2013). Cancer incidence and mortality patterns inEurope: estimates for 40 countries in 2012. Eur. J. Cancer.

[4] Chambers AF, Groom AC, MacDonald IC (2002). Dissemination and growth of cancer cells in metastatic sites. Nat. Rev. Cancer.

[5] Weigelt B, Peterse JL, van’t Veer LJ (2005). Breast cancer metastasis: markers and models. Nat. Rev. Cancer.

[6] Berman AT, Thukral AD, Hwang WT, Solin LJ, Vapiwala N (2013). Incidence and patterns of distant metastases for patients with early-stage breast cancer after breast conservation treatment. Clin. Breast Cancer.

[7] Luga V, Wrana JL (2013). Tumor-stroma interaction: revealing fibroblast-secreted exosomes as potent regulators of Wnt-planar cell polarity signaling in cancer metastasis. Cancer Res..

[8] Fantozzi A, Christofori G (2006). Mouse models of breast cancer metastasis. Breast Cancer Res..

[9] Fitamant J (2008). Netrin-1 expression confers a selective advantage for tumor cell survival in metastatic breast cancer. Proc. Natl Acad. Sci. USA.

[10] Morris DC (2017). Nck deficiency is associated with delayed breast carcinoma progression and reduced metastasis. Mol. Biol. Cell.

[11] Gengenbacher N, Singhal M, Augustin HG (2017). Preclinical mouse solid tumour models: status quo, challenges and perspectives. Nat. Rev. Cancer.

[12] Talmadge JE, Singh RK, Fidler IJ, Raz A (2007). Murine models to evaluate novel and conventional therapeutic strategies for cancer. Am. J. Pathol..

[13] Rashid OM (2013). Is tail vein injection a relevant breast cancer lung metastasis model?. J. Thorac. Dis..

[14] Lin S, Gregory RI (2015). MicroRNA biogenesis pathways in cancer. Nat. Rev. Cancer.

[15] Garzon R, Marcucci G, Croce CM (2010). Targeting microRNAs in cancer: rationale, strategies and challenges. Nat. Rev. Drug Discov..

[16] Yang S (2013). MicroRNA-34 suppresses breast cancer invasion and metastasis by directly targeting Fra-1. Oncogene.

[17] Baek D (2008). The impact of microRNAs on protein output. Nature.

[18] Guo H, Ingolia NT, Weissman JS, Bartel DP (2010). Mammalian microRNAs predominantly act to decrease target mRNA levels. Nature.

[19] Nielsen CB (2007). Determinants of targeting by endogenous and exogenous microRNAs and siRNAs. RNA.

[20] Selbach M (2008). Widespread changes in protein synthesis induced by microRNAs. Nature.

[21] Pillar N (2017). MicroRNAs as predictors for CNS relapse of systemic diffuse large B-cell lymphoma. Oncotarget.

[22] Love MI, Huber W, Anders S (2014). Moderated estimation of fold change and dispersion for RNA-seq data with DESeq2. Genome Biol..

[23] Lowry MC, Gallagher WM, O’Driscoll L (2015). The role of exosomes in breast cancer. Clin. Chem..

[24] Gilam A (2016). Local microRNA delivery targets Palladin and prevents metastatic breast cancer. Nat. Commun..

[25] Tian Y (2016). Expression of ATP binding cassette E1 enhances viability and invasiveness of lung adenocarcinoma cells in vitro. Mol. Med. Rep..

[26] Tiscornia G, Singer O, Verma IM (2006). Design and cloning of lentiviral vectors expressing small interfering RNAs. Nat. Protoc..

[27] Oved K (2017). MicroRNA-mediated regulation of ITGB3 and CHL1 is implicated in SSRI action. Front. Mol. Neurosci..

[28] Anders S, Pyl PT, Huber W (2015). HTSeq—a Python framework to work with high-throughput sequencing data. Bioinformatics.

[29] Gilam A (2017). MicroRNA regulation of progesterone receptor in breast cancer. Oncotarget.

[30] Timmins NE, Harding FJ, Smart C, Brown MA, Nielsen LK (2005). Method for the generation and cultivation of functional three-dimensional mammary constructs without exogenous extracellular matrix. Cell Tissue Res..

[31] Paraskevopoulou MD (2013). DIANA-microT web serverv5.0: service integration into miRNA functional analysis workflows. Nucleic Acids Res..

[32] Betel D, Koppal A, Agius P, Sander C, Leslie C (2010). Comprehensive modeling of microRNA targets predicts functional non-conserved and non-canonical sites. Genome Biol..

[33] Wang X (2016). Improving microRNA target prediction by modeling with unambiguously identified microRNA-target pairs from CLIP-ligation studies. Bioinform. Oxf. Engl..

[34] Vejnar CE, Zdobnov EM (2012). MiRmap: comprehensive prediction of microRNA target repression strength. Nucleic Acids Res..

[35] Hsu SD (2008). miRNAMap 2.0: genomic maps of microRNAs in metazoan genomes. Nucleic Acids Res..

[36] Anders G (2012). doRiNA: a database of RNA interactions in post-transcriptional regulation. Nucleic Acids Res..

[37] Kertesz M, Iovino N, Unnerstall U, Gaul U, Segal E (2007). The role of site accessibility in microRNA target recognition. Nat. Genet..

[38] Miranda KC (2006). A pattern-based method for the identification of MicroRNA binding sites and their corresponding heteroduplexes. Cell.

[39] Krüger J, Rehmsmeier M (2006). RNAhybrid: microRNA target prediction easy, fast and flexible. Nucleic Acids Res..

[40] Friedman RC, Farh KKH, Burge CB, Bartel DP (2009). Most mammalian mRNAs are conserved targets of microRNAs. Genome Res..

[41] Rani J, Shah ABR, Ramachandran S (2015). pubmed.mineR: an R package with text-mining algorithms to analyse PubMed abstracts. J. Biosci..

[42] Uhlén M (2015). Proteomics. Tissue-based map of the human proteome. Science.

[43] Lánczky A (2016). miRpower: a web-tool to validate survival-associated miRNAs utilizing expression data from 2178 breast cancer patients. Breast Cancer Res. Treat..

[44] Pereira Bernard, Chin Suet-Feung, Rueda Oscar M., Vollan Hans-Kristian Moen, Provenzano Elena, Bardwell Helen A., Pugh Michelle, Jones Linda, Russell Roslin, Sammut Stephen-John, Tsui Dana W. Y., Liu Bin, Dawson Sarah-Jane, Abraham Jean, Northen Helen, Peden John F., Mukherjee Abhik, Turashvili Gulisa, Green Andrew R., McKinney Steve, Oloumi Arusha, Shah Sohrab, Rosenfeld Nitzan, Murphy Leigh, Bentley David R., Ellis Ian O., Purushotham Arnie, Pinder Sarah E., Børresen-Dale Anne-Lise, Earl Helena M., Pharoah Paul D., Ross Mark T., Aparicio Samuel, Caldas Carlos (2016). The somatic mutation profiles of 2,433 breast cancers refines their genomic and transcriptomic landscapes. Nature Communications.

[45] Gao J (2013). Integrative analysis of complex cancer genomics and clinical profiles using the cBioPortal. Sci. Signal..

[46] Chou CH (2016). miRTarBase 2016: updates to the experimentally validated miRNA-target interactions database. Nucleic Acids Res..

[47] Yu G, Wang LG, Han Y, He QY (2012). clusterProfiler: an R package for comparing biological themes among gene clusters. Omics J. Integr. Biol..

[48] Lambert AW, Pattabiraman DR, Weinberg RA (2017). Emerging biological principles of metastasis. Cell.

[49] Hong Y (2016). miR-96 promotes cell proliferation, migration and invasion by targeting PTPN9 in breast cancer. Sci. Rep..

[50] Li P (2014). MiR-183/-96/-182 cluster is up-regulated in most breast cancers and increases cell proliferation and migration. Breast Cancer Res..

[51] Wang Y, Huang JW, Calses P, Kemp CJ, Taniguchi T (2012). MiR-96 downregulates REV1 and RAD51 to promote cellular sensitivity to cisplatin and PARP inhibition. Cancer Res..

[52] Li C (2014). GPC1 regulated by miR-96-5p, rather than miR-182-5p, in inhibition of pancreatic carcinoma cell proliferation. Int. J. Mol. Sci..

[53] Baik SH, Lee J, Lee YS, Jang JY, Kim CW (2016). ANT2 shRNA downregulates miR-19a and miR-96 through the PI3K/Akt pathway and suppresses tumor growth in hepatocellular carcinoma cells. Exp. Mol. Med..

[54] Hlaváč V (2013). The expression profile of ATP-binding cassette transporter genes in breast carcinoma. Pharmacogenomics.

[55] Han X, Tian Y, Tian D (2016). Tumor metastatic promoter ABCE1 interacts with the cytoskeleton protein actin and increases cell motility. Oncol. Rep..

[56] Huang B, Zhou H, Lang X, Liu Z (2014). siRNA-induced ABCE1 silencing inhibits proliferation and invasion of breast cancer cells. Mol. Med. Rep..

[57] Reynolds DS (2017). Breast cancer spheroids reveal a differential cancer stem cell Response to chemotherapeutic treatment. Sci. Rep..

[58] Erez N, Truitt M, Olson P, Hanahan D (2010). Cancer-associated fibroblasts are activated in incipient neoplasia to orchestrate tumor-promoting inflammation in an NF-κB-dependent manner. Cancer Cell.

[59] Zhao L (2012). MiRNA expression analysis of cancer-associated fibroblasts and normal fibroblasts in breast cancer. Int. J. Biochem. Cell Biol..

[60] Saleh SMI (2017). Identification of interacting stromal axes in triple-negative breast cancer. Cancer Res..

