# Supplementary figures and images for: Comparison of breast cancer metastasis models reveals a possible mechanism of tumor aggressiveness
Source: Cell Death Dis. 2018 Oct 10;9(10):1040. doi: 10.1038/s41419-018-1094-8 (PMC6180100; doi:10.1038/s41419-018-1094-8)

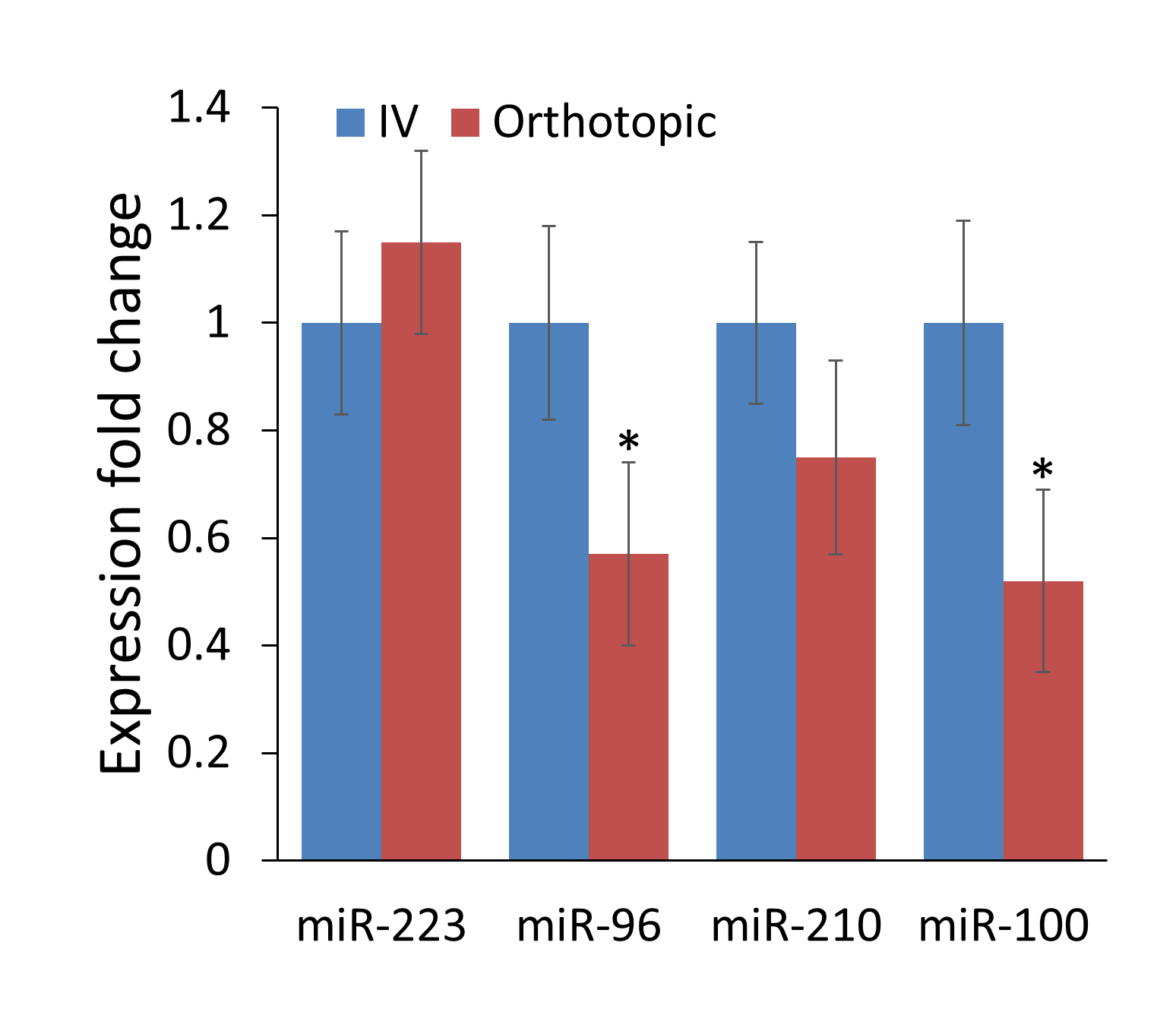

Supplement: Supplementary file 1 — Supplementary figure 1 [file 41419_2018_1094_MOESM1_ESM.tif]
