# Supplementary material for: Comparison of breast cancer metastasis models reveals a possible mechanism of tumor aggressiveness
Source: Cell Death Dis. 2018 Oct 10;9(10):1040. doi: 10.1038/s41419-018-1094-8 (PMC6180100; doi:10.1038/s41419-018-1094-8)
Supplement: Supplementary file 2 — Caption for supplementary figure 1 [file 41419_2018_1094_MOESM2_ESM.docx]

Supplementary figure 1- miR-223, miR-96, miR-210, and miR-100 relative expression in validation experiment

qPCR analysis of RNA extracted from lung macrometastases (two macrometastases were taken from each mouse in the IV and orthotopic groups, n=3) revealed that miR-96 and miR-100 were significantly downregulated in the orthotopic group compared to the IV group. miR-223 and miR-210 expression levels were not significantly different between treatment groups. Data is presented as mean +/- SEM. *<0.05
