# Supplementary material for: Comparison of breast cancer metastasis models reveals a possible mechanism of tumor aggressiveness
Source: Cell Death Dis. 2018 Oct 10;9(10):1040. doi: 10.1038/s41419-018-1094-8 (PMC6180100; doi:10.1038/s41419-018-1094-8)
Supplement: Supplementary file 3 — Supplementary table 1 [file 41419_2018_1094_MOESM3_ESM.docx]

**Supplementary table 1. SYBR green RT-PCR primers for mRNA quantification**

| **Primer name** | **Sequence** |
| --- | --- |
| M Abce1 For | AGCTGGAGAGTTCACGGACTCTGA |
| M Abce1 Rev | AAGCCTTCCAGCAAGCATTCTG |
| H ABCE1 For | TTGCTTGTGCTGTCGTTTG |
| H ABCE1 Rev | AGAGATCGTATAGTAATAGCAGCCTT |
| M β-Actin-For | ACCAGAGGCATACAGGGACA |
| M β-Actin-Rev | CTAAGGCCAACCGTGAAAAG |
| H GAPDH-For | AGCCACATCGCTGAGACA |
| H GAPDH-Rev | GCCCAATACGACCAAATCC |

* H-human, M-mouse
