# Supplementary material for: Comparison of breast cancer metastasis models reveals a possible mechanism of tumor aggressiveness
Source: Cell Death Dis. 2018 Oct 10;9(10):1040. doi: 10.1038/s41419-018-1094-8 (PMC6180100; doi:10.1038/s41419-018-1094-8)
Supplement: Supplementary file 4 — Supplementary table 2 [file 41419_2018_1094_MOESM4_ESM.docx]

**Supplementary table 2. Primary antibodies used for Western blot analysis**

| **Antibody** | **Manufacturer** | **Catalog number** | **Dilution** |
| --- | --- | --- | --- |
| Rabbit anti-ABCE1 | Abcam | Ab32270 | 1:1000 |
| Rabbit anti-ABCE1 | Santa-Cruz | Sc-133219 | 1:1000 |
| Mouse anti-ACTIN | Millipore | clone C4, MAB1501 | 1:5000 |
