# Supplementary material for: Comparison of breast cancer metastasis models reveals a possible mechanism of tumor aggressiveness
Source: Cell Death Dis. 2018 Oct 10;9(10):1040. doi: 10.1038/s41419-018-1094-8 (PMC6180100; doi:10.1038/s41419-018-1094-8)
Supplement: Supplementary file 5 — Supplementary table 3 [file 41419_2018_1094_MOESM5_ESM.docx]

**Supplementary table 3. Secondary antibodies used for Western blot analysis**

| **Antibody** | **Manufacturer** | **Catalog number** | **Dilution** |
| --- | --- | --- | --- |
| Rabbit on Rodent HRP-polymer | Biocare Medical, Inc. | RMR 622 | 1:10000 |
| HRP-conjugated AffiniPure Goat-anti-Mouse IgG | Jackson ImmunoResearch Laboratories, Inc. | 115-035-166 | 1:10000 |
